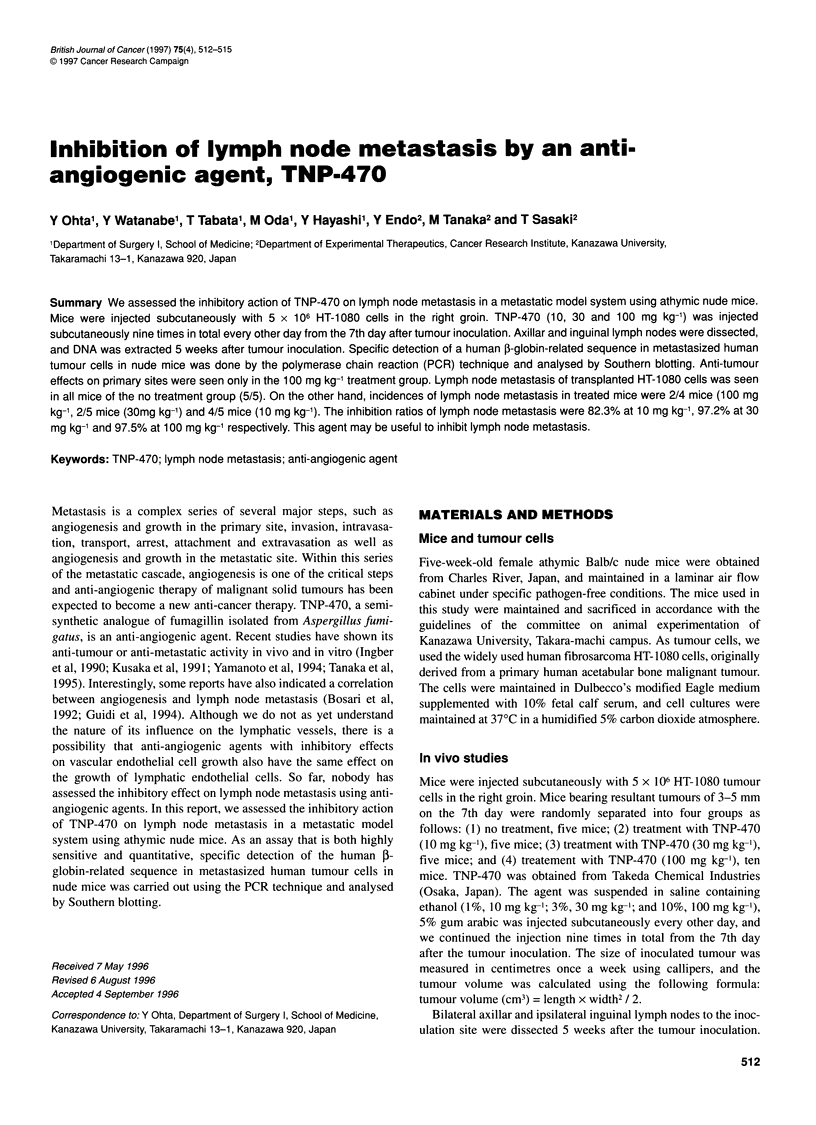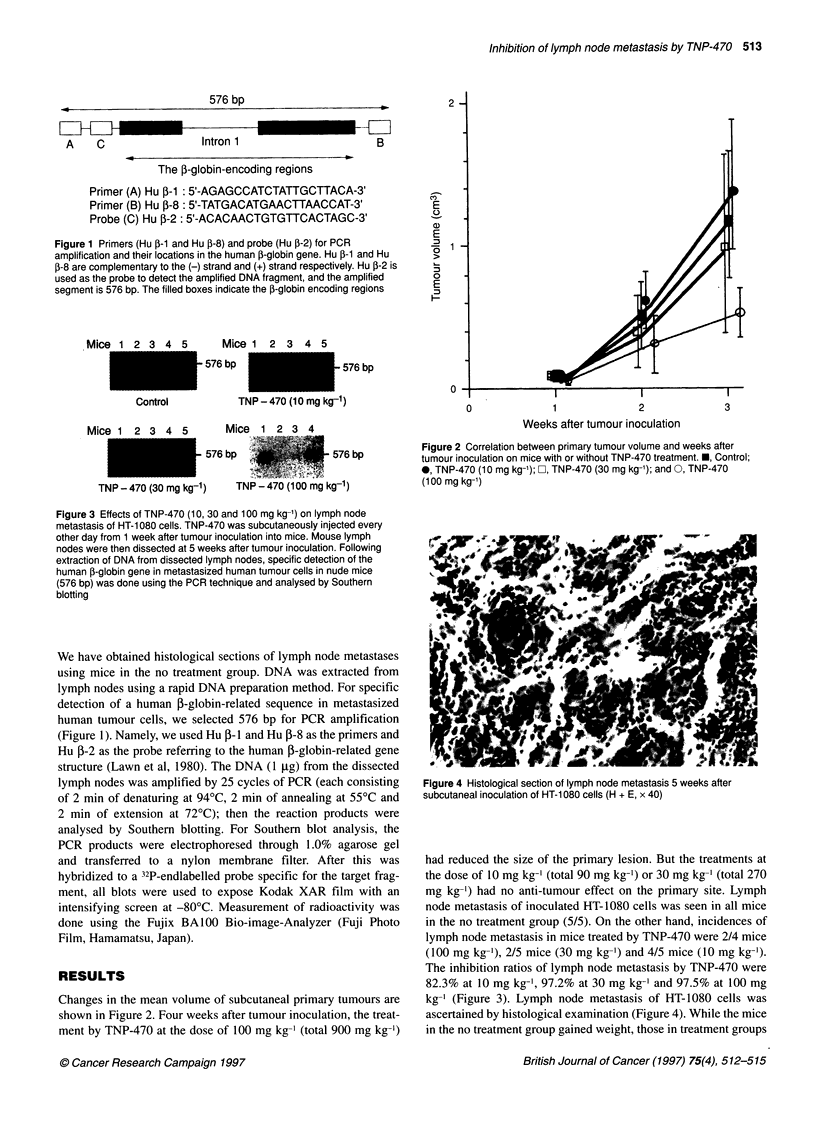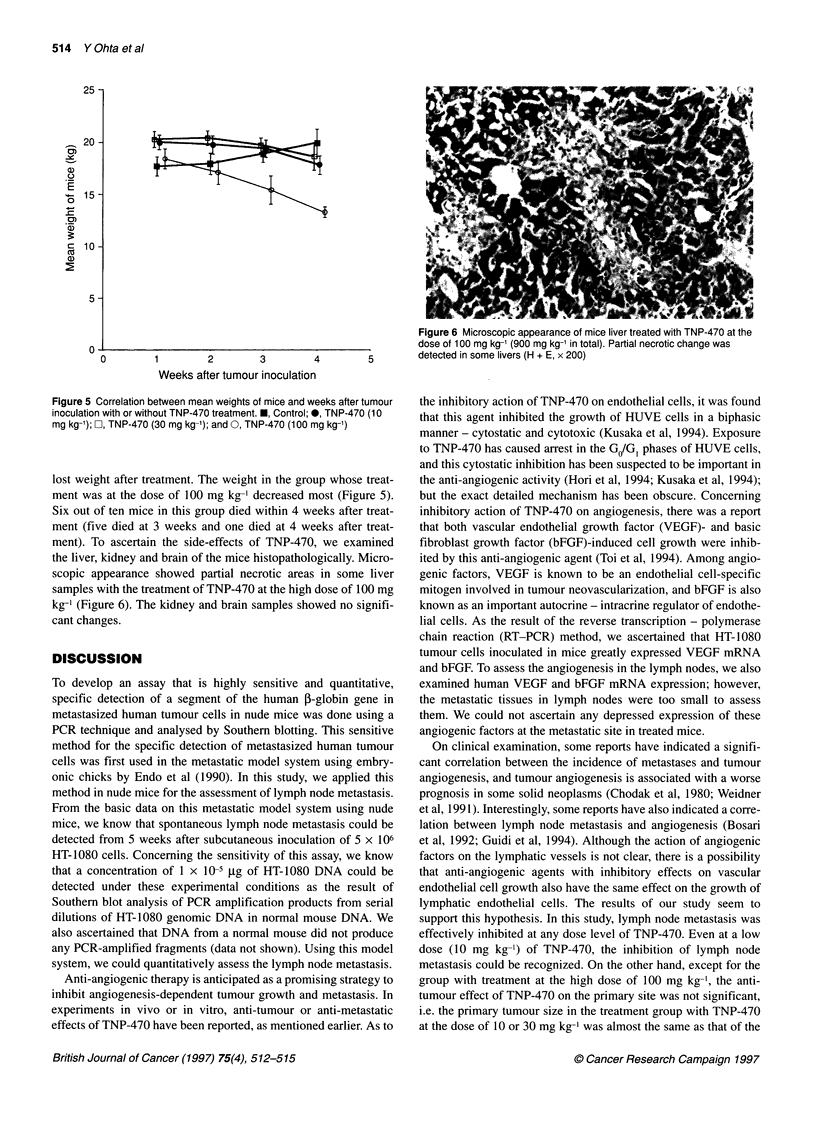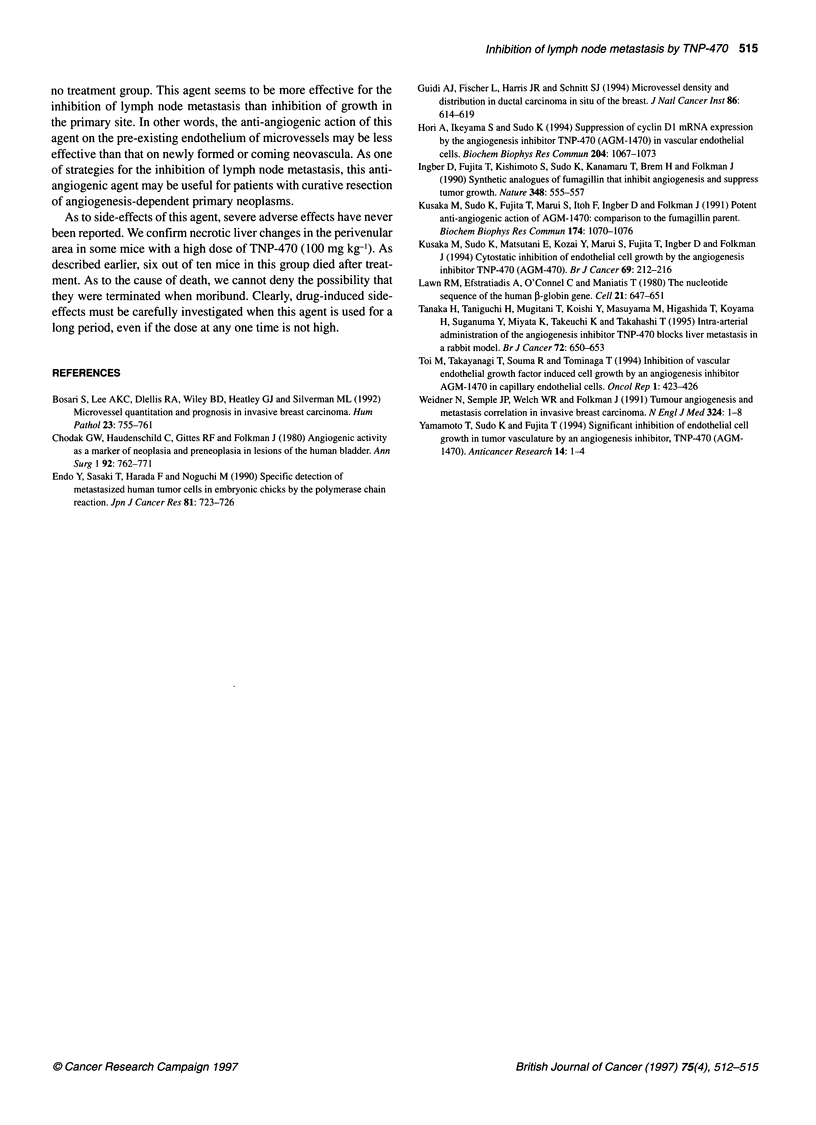# Inhibition of lymph node metastasis by an anti-angiogenic agent, TNP-470.

**DOI:** 10.1038/bjc.1997.89

**Published:** 1997

**Authors:** Y. Ohta, Y. Watanabe, T. Tabata, M. Oda, Y. Hayashi, Y. Endo, M. Tanaka, T. Sasaki

**Affiliations:** Department of Surgery I, School of Medicine, Kanazawa University, Japan.

## Abstract

**Images:**


British Journal of Cancer (1997) 75(4), 512–515
© 1997 Cancer Research Campaign

### Inhibition of lymph node metastasis by an anti-angiogenic agent, TNP-470

**Y Ohta[1], Y Watanabe[1], T Tabata[1], M Oda[1], Y Hayashi[1], Y Endo[2], M Tanaka[2] and T Sasaki[2]**

[1]Department of Surgery I, School of Medicine; [2]Department of Experimental Therapeutics, Cancer Research Institute, Kanazawa University, Takaramachi 13–1, Kanazawa 920, Japan

**Summary** We assessed the inhibitory action of TNP-470 on lymph node metastasis in a metastatic model system using athymic nude mice. Mice were injected subcutaneously with $5 \times 10^6$ HT-1080 cells in the right groin. TNP-470 (10, 30 and 100 mg $kg^{-1}$) was injected subcutaneously nine times in total every other day from the 7th day after tumour inoculation. Axillar and inguinal lymph nodes were dissected, and DNA was extracted 5 weeks after tumour inoculation. Specific detection of a human β-globin-related sequence in metastasized human tumour cells in nude mice was done by the polymerase chain reaction (PCR) technique and analysed by Southern blotting. Anti-tumour effects on primary sites were seen only in the 100 mg $kg^{-1}$ treatment group. Lymph node metastasis of transplanted HT-1080 cells was seen in all mice of the no treatment group (5/5). On the other hand, incidences of lymph node metastasis in treated mice were 2/4 mice (100 mg $kg^{-1}$, 2/5 mice (30mg $kg^{-1}$) and 4/5 mice (10 mg $kg^{-1}$). The inhibition ratios of lymph node metastasis were 82.3% at 10 mg $kg^{-1}$, 97.2% at 30 mg $kg^{-1}$ and 97.5% at 100 mg $kg^{-1}$ respectively. This agent may be useful to inhibit lymph node metastasis.

**Keywords:** TNP-470; lymph node metastasis; anti-angiogenic agent

Metastasis is a complex series of several major steps, such as angiogenesis and growth in the primary site, invasion, intravasation, transport, arrest, attachment and extravasation as well as angiogenesis and growth in the metastatic site. Within this series of the metastatic cascade, angiogenesis is one of the critical steps and anti-angiogenic therapy of malignant solid tumours has been expected to become a new anti-cancer therapy. TNP-470, a semi-synthetic analogue of fumagillin isolated from *Aspergillus fumigatus*, is an anti-angiogenic agent. Recent studies have shown its anti-tumour or anti-metastatic activity in vivo and in vitro (Ingber et al, 1990; Kusaka et al, 1991; Yamanoto et al, 1994; Tanaka et al, 1995). Interestingly, some reports have also indicated a correlation between angiogenesis and lymph node metastasis (Bosari et al, 1992; Guidi et al, 1994). Although we do not as yet understand the nature of its influence on the lymphatic vessels, there is a possibility that anti-angiogenic agents with inhibitory effects on vascular endothelial cell growth also have the same effect on the growth of lymphatic endothelial cells. So far, nobody has assessed the inhibitory effect on lymph node metastasis using anti-angiogenic agents. In this report, we assessed the inhibitory action of TNP-470 on lymph node metastasis in a metastatic model system using athymic nude mice. As an assay that is both highly sensitive and quantitative, specific detection of the human β-globin-related sequence in metastasized human tumour cells in nude mice was carried out using the PCR technique and analysed by Southern blotting.

*Received 7 May 1996*
*Revised 6 August 1996*
*Accepted 4 September 1996*

*Correspondence to:* Y Ohta, Department of Surgery I, School of Medicine, Kanazawa University, Takaramachi 13–1, Kanazawa 920, Japan

### MATERIALS AND METHODS

### Mice and tumour cells

Five-week-old female athymic Balb/c nude mice were obtained from Charles River, Japan, and maintained in a laminar air flow cabinet under specific pathogen-free conditions. The mice used in this study were maintained and sacrificed in accordance with the guidelines of the committee on animal experimentation of Kanazawa University, Takara-machi campus. As tumour cells, we used the widely used human fibrosarcoma HT-1080 cells, originally derived from a primary human acetabular bone malignant tumour. The cells were maintained in Dulbecco's modified Eagle medium supplemented with 10% fetal calf serum, and cell cultures were maintained at 37°C in a humidified 5% carbon dioxide atmosphere.

### In vivo studies

Mice were injected subcutaneously with $5 \times 10^6$ HT-1080 tumour cells in the right groin. Mice bearing resultant tumours of 3–5 mm on the 7th day were randomly separated into four groups as follows: (1) no treatment, five mice; (2) treatment with TNP-470 (10 mg $kg^{-1}$), five mice; (3) treatment with TNP-470 (30 mg $kg^{-1}$), five mice; and (4) treatement with TNP-470 (100 mg $kg^{-1}$), ten mice. TNP-470 was obtained from Takeda Chemical Industries (Osaka, Japan). The agent was suspended in saline containing ethanol (1%, 10 mg $kg^{-1}$; 3%, 30 mg $kg^{-1}$; and 10%, 100 mg $kg^{-1}$), 5% gum arabic was injected subcutaneously every other day, and we continued the injection nine times in total from the 7th day after the tumour inoculation. The size of inoculated tumour was measured in centimetres once a week using callipers, and the tumour volume was calculated using the following formula: tumour volume ($cm^3$) = length × $width^2$ / 2.

Bilateral axillar and ipsilateral inguinal lymph nodes to the inoculation site were dissected 5 weeks after the tumour inoculation.

**Figure 1** Primers (Hu β-1 and Hu β-8) and probe (Hu β-2) for PCR amplification and their locations in the human β-globin gene. Hu β-1 and Hu β-8 are complementary to the (–) strand and (+) strand respectively. Hu β-2 is used as the probe to detect the amplified DNA fragment, and the amplified segment is 576 bp. The filled boxes indicate the β-globin encoding regions

**Figure 3** Effects of TNP-470 (10, 30 and 100 mg kg$^{-1}$) on lymph node metastasis of HT-1080 cells. TNP-470 was subcutaneously injected every other day from 1 week after tumour inoculation into mice. Mouse lymph nodes were then dissected at 5 weeks after tumour inoculation. Following extraction of DNA from dissected lymph nodes, specific detection of the human β-globin gene in metastasized human tumour cells in nude mice (576 bp) was done using the PCR technique and analysed by Southern blotting

**Figure 2** Correlation between primary tumour volume and weeks after tumour inoculation on mice with or without TNP-470 treatment. ■, Control; ●, TNP-470 (10 mg kg$^{-1}$); □, TNP-470 (30 mg kg$^{-1}$); and ○, TNP-470 (100 mg kg$^{-1}$)

**Figure 4** Histological section of lymph node metastasis 5 weeks after subcutaneal inoculation of HT-1080 cells (H + E, × 40)

We have obtained histological sections of lymph node metastases using mice in the no treatment group. DNA was extracted from lymph nodes using a rapid DNA preparation method. For specific detection of a human β-globin-related sequence in metastasized human tumour cells, we selected 576 bp for PCR amplification (Figure 1). Namely, we used Hu β-1 and Hu β-8 as the primers and Hu β-2 as the probe referring to the human β-globin-related gene structure (Lawn et al, 1980). The DNA (1 μg) from the dissected lymph nodes was amplified by 25 cycles of PCR (each consisting of 2 min of denaturing at 94°C, 2 min of annealing at 55°C and 2 min of extension at 72°C); then the reaction products were analysed by Southern blotting. For Southern blot analysis, the PCR products were electrophoresed through 1.0% agarose gel and transferred to a nylon membrane filter. After this was hybridized to a $^{32}$P-endlabelled probe specific for the target fragment, all blots were used to expose Kodak XAR film with an intensifying screen at –80°C. Measurement of radioactivity was done using the Fujix BA100 Bio-image-Analyzer (Fuji Photo Film, Hamamatsu, Japan).

### RESULTS

Changes in the mean volume of subcutaneal primary tumours are shown in Figure 2. Four weeks after tumour inoculation, the treatment by TNP-470 at the dose of 100 mg kg$^{-1}$ (total 900 mg kg$^{-1}$) had reduced the size of the primary lesion. But the treatments at the dose of 10 mg kg$^{-1}$ (total 90 mg kg$^{-1}$) or 30 mg kg$^{-1}$ (total 270 mg kg$^{-1}$) had no anti-tumour effect on the primary site. Lymph node metastasis of inoculated HT-1080 cells was seen in all mice in the no treatment group (5/5). On the other hand, incidences of lymph node metastasis in mice treated by TNP-470 were 2/4 mice (100 mg kg$^{-1}$), 2/5 mice (30 mg kg$^{-1}$) and 4/5 mice (10 mg kg$^{-1}$). The inhibition ratios of lymph node metastasis by TNP-470 were 82.3% at 10 mg kg$^{-1}$, 97.2% at 30 mg kg$^{-1}$ and 97.5% at 100 mg kg$^{-1}$ (Figure 3). Lymph node metastasis of HT-1080 cells was ascertained by histological examination (Figure 4). While the mice in the no treatment group gained weight, those in treatment groups

*© Cancer Research Campaign 1997*

**Figure 5** Correlation between mean weights of mice and weeks after tumour inoculation with or without TNP-470 treatment. ■, Control; ●, TNP-470 (10 mg kg$^{-1}$); □, TNP-470 (30 mg kg$^{-1}$); and ○, TNP-470 (100 mg kg$^{-1}$)

**Figure 6** Microscopic appearance of mice liver treated with TNP-470 at the dose of 100 mg kg$^{-1}$ (900 mg kg$^{-1}$ in total). Partial necrotic change was detected in some livers (H + E, × 200)

lost weight after treatment. The weight in the group whose treatment was at the dose of 100 mg kg$^{-1}$ decreased most (Figure 5). Six out of ten mice in this group died within 4 weeks after treatment (five died at 3 weeks and one died at 4 weeks after treatment). To ascertain the side-effects of TNP-470, we examined the liver, kidney and brain of the mice histopathologically. Microscopic appearance showed partial necrotic areas in some liver samples with the treatment of TNP-470 at the high dose of 100 mg kg$^{-1}$ (Figure 6). The kidney and brain samples showed no significant changes.

### DISCUSSION

To develop an assay that is highly sensitive and quantitative, specific detection of a segment of the human β-globin gene in metastasized human tumour cells in nude mice was done using a PCR technique and analysed by Southern blotting. This sensitive method for the specific detection of metastasized human tumour cells was first used in the metastatic model system using embryonic chicks by Endo et al (1990). In this study, we applied this method in nude mice for the assessment of lymph node metastasis. From the basic data on this metastatic model system using nude mice, we know that spontaneous lymph node metastasis could be detected from 5 weeks after subcutaneous inoculation of $5 \times 10^6$ HT-1080 cells. Concerning the sensitivity of this assay, we know that a concentration of $1 \times 10^{-5}$ μg of HT-1080 DNA could be detected under these experimental conditions as the result of Southern blot analysis of PCR amplification products from serial dilutions of HT-1080 genomic DNA in normal mouse DNA. We also ascertained that DNA from a normal mouse did not produce any PCR-amplified fragments (data not shown). Using this model system, we could quantitatively assess the lymph node metastasis.

Anti-angiogenic therapy is anticipated as a promising strategy to inhibit angiogenesis-dependent tumour growth and metastasis. In experiments in vivo or in vitro, anti-tumour or anti-metastatic effects of TNP-470 have been reported, as mentioned earlier. As to the inhibitory action of TNP-470 on endothelial cells, it was found that this agent inhibited the growth of HUVE cells in a biphasic manner – cytostatic and cytotoxic (Kusaka et al, 1994). Exposure to TNP-470 has caused arrest in the $G_0/G_1$ phases of HUVE cells, and this cytostatic inhibition has been suspected to be important in the anti-angiogenic activity (Hori et al, 1994; Kusaka et al, 1994); but the exact detailed mechanism has been obscure. Concerning inhibitory action of TNP-470 on angiogenesis, there was a report that both vascular endothelial growth factor (VEGF)- and basic fibroblast growth factor (bFGF)-induced cell growth were inhibited by this anti-angiogenic agent (Toi et al, 1994). Among angiogenic factors, VEGF is known to be an endothelial cell-specific mitogen involved in tumour neovascularization, and bFGF is also known as an important autocrine – intracrine regulator of endothelial cells. As the result of the reverse transcription – polymerase chain reaction (RT–PCR) method, we ascertained that HT-1080 tumour cells inoculated in mice greatly expressed VEGF mRNA and bFGF. To assess the angiogenesis in the lymph nodes, we also examined human VEGF and bFGF mRNA expression; however, the metastatic tissues in lymph nodes were too small to assess them. We could not ascertain any depressed expression of these angiogenic factors at the metastatic site in treated mice.

On clinical examination, some reports have indicated a significant correlation between the incidence of metastases and tumour angiogenesis, and tumour angiogenesis is associated with a worse prognosis in some solid neoplasms (Chodak et al, 1980; Weidner et al, 1991). Interestingly, some reports have also indicated a correlation between lymph node metastasis and angiogenesis (Bosari et al, 1992; Guidi et al, 1994). Although the action of angiogenic factors on the lymphatic vessels is not clear, there is a possibility that anti-angiogenic agents with inhibitory effects on vascular endothelial cell growth also have the same effect on the growth of lymphatic endothelial cells. The results of our study seem to support this hypothesis. In this study, lymph node metastasis was effectively inhibited at any dose level of TNP-470. Even at a low dose (10 mg kg$^{-1}$) of TNP-470, the inhibition of lymph node metastasis could be recognized. On the other hand, except for the group with treatment at the high dose of 100 mg kg$^{-1}$, the anti-tumour effect of TNP-470 on the primary site was not significant, i.e. the primary tumour size in the treatment group with TNP-470 at the dose of 10 or 30 mg kg$^{-1}$ was almost the same as that of the

© Cancer Research Campaign 1997

no treatment group. This agent seems to be more effective for the inhibition of lymph node metastasis than inhibition of growth in the primary site. In other words, the anti-angiogenic action of this agent on the pre-existing endothelium of microvessels may be less effective than that on newly formed or coming neovascula. As one of strategies for the inhibition of lymph node metastasis, this anti-angiogenic agent may be useful for patients with curative resection of angiogenesis-dependent primary neoplasms.

As to side-effects of this agent, severe adverse effects have never been reported. We confirm necrotic liver changes in the perivenular area in some mice with a high dose of TNP-470 (100 mg $kg^{-1}$). As described earlier, six out of ten mice in this group died after treatment. As to the cause of death, we cannot deny the possibility that they were terminated when moribund. Clearly, drug-induced side-effects must be carefully investigated when this agent is used for a long period, even if the dose at any one time is not high.

### REFERENCES

Bosari S, Lee AKC, Dlellis RA, Wiley BD, Heatley GJ and Silverman ML (1992) Microvessel quantitation and prognosis in invasive breast carcinoma. *Hum Pathol* **23**: 755–761

Chodak GW, Haudenschild C, Gittes RF and Folkman J (1980) Angiogenic activity as a marker of neoplasia and preneoplasia in lesions of the human bladder. *Ann Surg* 1 **92**: 762–771

Endo Y, Sasaki T, Harada F and Noguchi M (1990) Specific detection of metastasized human tumor cells in embryonic chicks by the polymerase chain reaction. *Jpn J Cancer Res* **81**: 723–726

Guidi AJ, Fischer L, Harris JR and Schnitt SJ (1994) Microvessel density and distribution in ductal carcinoma in situ of the breast. *J Natl Cancer Inst* **86**: 614–619

Hori A, Ikeyama S and Sudo K (1994) Suppression of cyclin D1 mRNA expression by the angiogenesis inhibitor TNP-470 (AGM-1470) in vascular endothelial cells. *Biochem Biophys Res Commun* **204**: 1067–1073

Ingber D, Fujita T, Kishimoto S, Sudo K, Kanamaru T, Brem H and Folkman J (1990) Synthetic analogues of fumagillin that inhibit angiogenesis and suppress tumor growth. *Nature* **348**: 555–557

Kusaka M, Sudo K, Fujita T, Marui S, Itoh F, Ingber D and Folkman J (1991) Potent anti-angiogenic action of AGM-1470: comparison to the fumagillin parent. *Biochem Biophys Res Commun* **174**: 1070–1076

Kusaka M, Sudo K, Matsutani E, Kozai Y, Marui S, Fujita T, Ingber D and Folkman J (1994) Cytostatic inhibition of endothelial cell growth by the angiogenesis inhibitor TNP-470 (AGM-470). *Br J Cancer* **69**: 212–216

Lawn RM, Efstratiadis A, O'Connel C and Maniatis T (1980) The nucleotide sequence of the human β-globin gene. *Cell* **21**: 647–651

Tanaka H, Taniguchi H, Mugitani T, Koishi Y, Masuyama M, Higashida T, Koyama H, Suganuma Y, Miyata K, Takeuchi K and Takahashi T (1995) Intra-arterial administration of the angiogenesis inhibitor TNP-470 blocks liver metastasis in a rabbit model. *Br J Cancer* **72**: 650–653

Toi M, Takayanagi T, Souma R and Tominaga T (1994) Inhibition of vascular endothelial growth factor induced cell growth by an angiogenesis inhibitor AGM-1470 in capillary endothelial cells. *Oncol Rep* **1**: 423–426

Weidner N, Semple JP, Welch WR and Folkman J (1991) Tumour angiogenesis and metastasis correlation in invasive breast carcinoma. *N Engl J Med* **324**: 1–8

Yamamoto T, Sudo K and Fujita T (1994) Significant inhibition of endothelial cell growth in tumor vasculature by an angiogenesis inhibitor, TNP-470 (AGM-1470). *Anticancer Research* **14**: 1–4

© Cancer Research Campaign 1997